# Event and Comment

**Published:** 1921-04

**Authors:** 


### DENTAL REGISTER

THE MAGAZINE OF DENTISTRY

Vol. LXXV APRIL, 1921 No. 4

### EVENT AND COMMENT

**Standardizing or Drifting**

In these days of new discoveries and changes of ideals and methods of practice, we sometimes wonder whether we are really getting wiser or only just changing ideals. Many of the old ideas have had to be abandoned because we have learned more scientific facts, and these facts, logically, have changed our methods of practice. But so many new facts are yet to be correlated or familiarized. We thought we had learned in general terms the nature and use of the science of bacteriology, but only to find that our ideas of sanitation have made a new medical practice imperative. Some of the older principles of technical dentistry are being changed to harmonize with present ideas of dental diseases, and the treatments of oral and systemic diseases in their relations to dental infections make radical changes necessary. Modern medical practice has made infection the ideal in the prevention of all medical and surgical practice. In a similar way these principles are being demanded in all dental and oral diseases. In fact the least we may say is that it is now believed that a careful scientific inspection of all dental lesions, or of any infection, demands careful scrutiny and preventive treatment before and after all technical operations. Our art is therefore only technically related to the fundamentally main objective of health. We can no longer strive for technical skill only, regardless of the health ideals that now force us to regard these as of vital importance.

In spite of such statements we are still dependent on our technical art to carry out needed artificial functions in the replacement of lost and crippled natural dentures. We rejoice that prosthetic advancement of marked character is going hand in hand with increasing knowledge of the needs for better dental health for the public generally.

The extraction of teeth goes on more rapidly than ever before, for most obvious reasons, and possibly this is to be deplored, but we shall no doubt in due time establish a safer system of treatment. We are probably extracting too many teeth now and our prosthesis may be unsafe and defective, but we shall learn to readjust our new notions to safer and more wholesome scientific formula and practices. At the present time patients and dentists are not willing to take so many chances with their teeth, because they fear the possibilities in neglect and the results of local and systemic consequences. We have swung from the habit of trying to save every tooth to that idea which now tells us that no tooth infection is to be tolerated on any account. It will take time and a lot of research to establish a safe and wholesome equilibrium, but we must use our wit and wisdom to do what seems best at the present until we come to some point of stability for action. What we need at the present time more than ever before is that we shall study more carefully our dental anatomy and pathology. Dentists do not know how to diagnose oral conditions as well as they should. We ought to know our patients' physiological conditions so well that we will be able to determine the degree of health in all superficial tissues at least, so that impending ill health may be suspected before it has become a serious systemic involvement. The time is past when a dentist can diagnose a serious health problem by the character of the pain in an individual tooth or even a suppurating alveolar process. Dental pains used to be local in character, but now we find that pain in any part of the body may be of the most serious consequences. We used to depend upon heat, cold, percussion and local pains of various degrees and kinds for making diagnoses of all cases. Then we made a grand improvement in the ability to discern by means of the radiograph that the alveolar environment in some conditions showed infection which enlarged our capacity for determining even the extent of the injuries or the disease. But we have yet to find out how far these local infections may be involved in systemic influences.

It is here that we are at present halting; not knowing whether we shall plunge into this state of imperfect knowledge of the science of medical art or revamp our system of dental education so that it will make it possible for the dental practitioner to begin anew the practices of dentistry as a medical scientist, or become associated as a scientific expert diagnostician. Can we safely go on practicing, even such a technical art, without a more comprehensive knowledge and skill short of a complete course of all medical art and science as we are now trying to do? Or is it practicable for a dentist to keep up the technical art and skill required for present-day practice, and gradually enlarge his therapeutic knowledge to such a degree that he can meet the present demands of the occasion, both technically and scientifically? There certainly will be a decided change in the method of practice if there is not an entirely new developing specialty of medicines or dentistry in the near future.

